# Supplementary material for: Leadership and management influences the outcome of wildlife reintroduction programs: findings from the Sea Eagle Recovery Project
Source: PeerJ. 2015 Jun 18;3:e1012. doi: 10.7717/peerj.1012 (PMC4476100; doi:10.7717/peerj.1012)
Supplement: Appendix S1 [file peerj-03-1012-s001.pdf]

APPENDIX A. Sample Interview Protocol.

First, I'd like to read you a statement. Please let me know if you consent:

PURPOSE: You are being asked to participate in a study on the organizational structure of wildlife reintroduction programs and its potential correlates to program performance. The purpose of this study is to understand whether the organizational characteristics of a wildlife reintroduction program can be related to the program's ultimate performance.

PROCEDURES: During this confidential interview, you will be asked to answer questions related to the reintroduction program in which you participated. The questions address elements of the planning, public relations, structure, and evaluation processes that took place within your program. The survey consists of 33 questions, and will take approximately 35 minutes to complete.

RISKS: I anticipate no risk from participating in this confidential survey.

BENEFITS: No benefit can be promised to you from your participation in this survey.

ALTERNATIVES: You have the alternative not to participate in this study. You may also choose to redact your consent to participate in this study at any time. Your desire not to participate in this study or your request to withdraw will have no adverse effects on you or your relationship with myself or Texas A&M University.

COSTS: There are no costs to participating in this study.

PAYMENT: There is no payment for participating in this study.

CONFIDENTIALITY: This interview is confidential. Upon completion of the survey, your results will be submitted to a secure account held by the Principal Investigator. Results will be aggregated and collectively analyzed.

RIGHT TO WITHDRAW: Your participation is voluntary; you have the right to withdraw or to skip any questions at any time.

OTHER PERTINENT INFORMATION: I will answer any questions you may have about the study. If you have any questions after we finish, please feel free to call or email me at any time. If you have any questions concerning your rights as a research participant, please contact the Texas A&M Office Of Research Compliance at +001 (979) 458-1467.

Interviewer Name: ______________________________
Date: ______________________________
Interview Number: ______________________________
Location of Interview: ______________________________
Interviewee Number: ______________________________

Place of Birth:

Place of Residence:

Length of Time Living in Country/Current Residence:

About Your Experiences with Wildlife

Do you encounter wildlife around your home? If so, how frequently? What are these encounters like?

Do you encounter white-tailed eagles around your workplace? If so, how frequently? What are these encounters like?

Had you ever studied white-tailed eagles prior to working with the reintroduction program?

Had you ever seen a white-tailed eagle prior to the reintroduction program? If so, how frequently? In what context?

Did you ever see a white-tailed eagle after you began working with the reintroduction program? If so, how frequently? In what context?

Prior to the WTE, had you ever worked on or participated in any reintroduction program?

During/after the WTE, did you work on or participate in any other reintroduction program?

About the Reintroduction Program

In which phases of the reintroduction have you been involved?
[Eagle: Phase 1 – Isle of Rum; Phase 2 – Wester Ross; Phase 3 – East Coast (Fife)]
[Condor: Phase 1 – Rescue/Capture & Captive Breeding; Phase 2 – Los Padres/Hopper Mountain; Phase 3 – Vermillion Cliffs; Phase 4 – Pinnacles & Today]

Who was your employer during each phase of the reintroduction in which you participated?

Who is your current employer?

What was your job title/position during each phase of the reintroduction in which you participated?

Did any other people hold your same position during your time working with the reintroduction?

To whom were you responsible while you held this position? What were their positions? Under which entities were they employed?

Were you ever responsible for any employees/volunteers while you held this position? What were their positions? Under which entities were they employed?

Approximately how many other people were employed to work on the reintroduction under your same entity?

Have you participated in or been present at any releases? If so, which ones?

About the Organizational Structure & Decision-Making Process

What was the organizational structure of the reintroduction program like? Has it changed since you began working on the reintroduction? If so, in what ways?

How many levels of management existed?

What was the highest level of management/responsibility?

What was the organizational culture of the reintroduction program like? Was it very hierarchical, or more team-oriented?

How were ultimate decisions, about the overall purpose and direction of the reintroduction program, made? By whom?

How were proximate decisions, about the day-to-day operation of the program made? By whom? Did statements of proximate goals exist for this reintroduction? If so, how were these goals set? By whom?

Did a mission statement/statement of ultimate goals exist for this reintroduction? Did it change?

How were responsibilities divided/assigned within the reintroduction program? By whom?

Did regular evaluations take place within the reintroduction? How frequently? By whom?

About Your Opinion on Evaluating Wildlife Reintroduction Programs

In your opinion, what would define a success in any wildlife reintroduction?

In your opinion, what would define a failure in any wildlife reintroduction?

By what measures do you believe that any wildlife reintroduction program can be evaluated? Biological? Social?

How would you evaluate the status of this reintroduction?

Do you believe this reintroduction could be improved? If so, in what ways?

Do you plan to work with this reintroduction in the future? If so, in what capacity?

Do you plan to work with any other reintroductions in the future? If so, in what capacity?

Is there anything else you'd like to add?

Thank you for your time!
